# Myd88 Signaling Is Involved in the Inflammatory Response in LPS-Induced Mouse Epididymitis and Bone-Marrow-Derived Dendritic Cells

**DOI:** 10.3390/ijms24097838

**Published:** 2023-04-25

**Authors:** Jin-Chuan Liu, Peng Wang, Qun-Xiong Zeng, Chen Yang, Minmin Lyu, Yanfeng Li, William Shu-Biu Yeung, Philip Chi-Ngong Chiu, Gerhard Haidl, Jean-Pierre Allam, Yong-Gang Duan

**Affiliations:** 1 Shenzhen Key Laboratory of Fertility Regulation, Center of Assisted Reproduction and Embryology, The University of Hong Kong-Shenzhen Hospital, Shenzhen 518053, China; 2 Department of Obstetrics and Gynaecology, LKS Faculty of Medicine, The University of Hong Kong, Hong Kong SAR, China; 3 Department of Urology, Daping Hospital, Army Medical University, Chongqing 400042, China; 4 Department of Andrology, Bonn University Hospital, Campus-Venusberg 1, 53127 Bonn, Germany

**Keywords:** epididymitis, *Myd88*, CRISPR-Cas9, dendritic cells, male infertility

## Abstract

Epididymitis is an epididymal inflammation that may lead to male infertility. Dendritic cells (DCs) and myeloid differentiation primary response gene 88 (Myd88) were associated with epididymitis in rodents. However, the functions of Myd88 on epididymal DCs remain unclear. This study investigated the role of Myd88 in DCs for epididymitis. The Myd88 signaling pathway, phenotypes of DC subsets, and cytokines were investigated in lipopolysaccharide (LPS)-induced epididymitis in mice. CRISPR-Cas9 was used to knockout *Myd88* in bone-marrow-derived dendritic cells (BMDCs) and immortalized mouse epididymal (DC2) cell line. In the vivo experiments, levels of the proinflammatory cytokines IL-1α, IL-6, IL-17A, TNF-α, IL-1β, MCP-1, and GM-CSF, mRNA for *MyD88* related genes, and the percentages of monocyte-derived DCs (Mo-DCs) were significantly elevated in mice with epididymitis. In the vitro experiments, LPS significantly promoted the apoptosis of BMDCs. In addition, the concentration of inflammatory cytokines in BMDCs and DC2s were increased in the LPS group, while decreasing after the knockout of Myd88. These findings indicate that *Myd88* on DCs is involved in the inflammation of epididymitis in mice, which may be a potential target for better strategies regarding the treatment of immunological male infertility.

## 1. Introduction

Epididymitis involves the inflammation of the epididymis, which is caused by both infectious and non-infectious factors [1]. Epididymitis in boys under 14 years of age may be associated with the reflux of urine into the ejaculatory ducts, or a part of a post-infectious syndrome. Epididymitis in sexually active males is mainly caused by chlamydia trachomatis and Neisseria gonorrhoeae, while infected urine retrograde flowing in the ejaculatory duct caused by prostatic hypertrophy is the most common cause in epididymitis in men over 35 years old [2]. Epididymitis generally presents as a gradual onset of posterior testicular pain, usually unilateral [3]. Additionally, epididymitis may cause chronic scrotal pain, and even subsequent fertility problems [4]. Epididymitis, the most common site of intrascrotal inflammation, is an important cause of male infertility compared with the prostate or seminal vesicles [5]. Since male infertility has become a significant public health issue, it is vital to understand the underlying mechanism of epididymitis [6].

A variety of immune cells have been proven to exist in the epididymis of rodents and humans, including DCs, macrophages (MΦ), T cells, and B cells [7,8,9,10,11]. DCs are a heterogeneous population of antigen-presenting cells that are crucial regulators of the immune response and of immunological tolerance in numerous organs composed of several subsets. Common dendritic cell progenitors (CDPs) derived from hematopoietic stem cells of bone marrow further differentiate into two major families: pre-plasmacytoid DCs (pDCs) and pre-classical DCs (cDCs) [12]. Pre-pDCs differentiate into pDC and circulate in the blood to repopulate pDCs in lymphoid or peripheral tissues. Most studies on the matter have revealed that pDCs mainly mediate in virus-based infections and product type I interferon [13,14,15]. Pre-cDCs can differentiate into two subsets: Batf3-dependent DCs (cDC1) and IRF4-dependent DCs (cDC2), which are the main resident DCs in non-draining lymphoid organs. cDC1, including resident CD8^+^ DCs and CD103^+^ DCs, are cells that share a common molecular marker, such as XCR1 and Toll-like receptor 3 (TLR3). cDC2s are described as CD8^−^CD11b^+^ DCs and migratory CD11b^+^ DCs. cDC2s are specialized for inducing CD4^+^ T cell-mediated immunity [16]. Moreover, a population named monocyte-derived DCs (Mo-DCs) is derived from monocytes during pathogen or sterile-induced inflammation [17]. These cells express classical DCs markers such as CD11c and MHC II. They can also play the role of antigen-presenting cells and induce an adapted immune response under inflammatory conditions. Therefore, they are also named “inflammatory DCs” [18,19]. Our previous study demonstrated that CD11c^+^IL-23^+^ inflammatory DCs were significantly elevated and promoted Th17 cell development in human chronic epididymitis [20]. However, the functions of DC subsets and the molecular pathways involved in the pathogenesis of epididymal infections, particularly in the onset of acute epididymitis, remain largely unexplored.

Lipopolysaccharide-induced epididymitis is a well-established mouse model of epididymitis used for investigating the pathogenic mechanism involved in the role of the Toll-like receptor 4 (TLR4) as the outer membrane of Gram-negative bacteria such as *E. coli* and the agonist of TLR4 [21]. Two pathways are involved in the TLR4-mediated response to LPS: the MYD88-dependent and MYD88-independent pathways [22]. *Myd88* is widely expressed in most immune cells, and it acts as a central node of the inflammatory response [23]. It is an essential precondition for many inflammatory signaling pathways and is involved in the signal transduction of Toll-like and IL-1 receptor family members [24]. *Myd88* is also required for pattern recognition and Th-cell polarization by DCs [25]. While the deletion of *Myd88* expression could suppress the inflammatory response, *Myd88* has become a novel therapeutic target for many diseases related to inflammation, such as chronic obstructive pulmonary disease (COPD) and atherosclerosis [26,27]. Our previous study found that the tissue damage and inflammatory reactions were rescued in *Myd88*^−^/^−^ uropathogenic *E. coli* (UPEC) epididymitis in mice compared to wild-type mice [28]. Whether the effect of *Myd88* knockout in DCs can relieve the inflammation is unclear. Furthermore, Myd88 signaling pathway can be activated by environmental pollution, which has been proved to have harmful effects on semen quality [29,30,31]. A previous study has shown that the presence of specific volatile organic compounds can impact the sperm parameters, such as sperm morphology, sperm motility, and sperm count [31]. Another study has found that the sperm concentration and count of humans are negatively correlated with the degree of exposure to particulate matter [30]. Thus, Myd88 signaling pathway appears to play a significant role in male fertility in the industrialized world.

In recent years, the technology of clustered regularly interspaced short palindromic repeats (CRISPR)/CRISPR associated protein 9 (Cas9) transfections has become a popular method for gene editing in mammalian cells [32,33]. CRISPR-Cas9 technology can perform gene editing by targeting specific genomic loci through defined guide RNA (gRNA) sequences [34]. This method has successfully edited the genome of various cells, including primary cells and cell lines in mice or humans [35]. In this study, we explored the role of *Myd88* in LPS-induced epididymitis and BMDCs through CRISPR-Cas9 technology. Our results show that the expression of MYD88 signaling is significantly enhanced in LPS-induced mouse epididymitis. *Myd88*-KO in BMDCs leads to decreased inflammatory factors, indicating a potential role of the MYD88 signaling pathway in DCs involved in the pathogenesis of epididymitis.

## 2. Results

### 2.1. Increased Level of MYD88 Signaling Pathway in LPS-Induced Mouse Epididymitis

**Figure 1:**
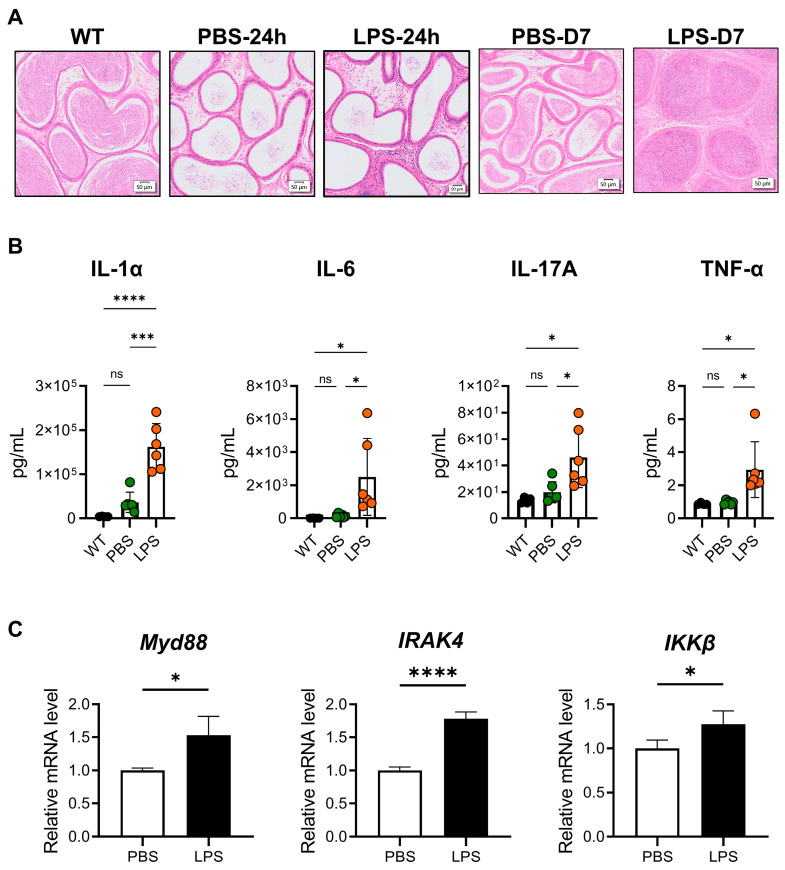
Increased levels of the MYD88 signaling pathway in LPS-induced mouse cauda epididymitis. The cauda epididymides were collected from 10–12-week-old C57BL/6J mice, with or without LPS treatment. (**A**) Histopathological analysis of the cauda epididymis in LPS-induced mouse cauda epididymitis. Representative micrographs of hematoxylin-eosin (HE)-stained epididymal sections from untreated control (WT), sham-treated controls (PBS), and LPS-induced mice, 24 h and 7 days after treatment, respectively (WT, *n* = 4; PBS and LPS, *n* = 6; scale bar = 50 μm). (**B**) Comparison of proinflammatory cytokines IL-1α, IL-6, IL-17A, and TNF-α among three groups at 24 h after LPS treatment. Results are expressed as mean ± SD and analyzed by one-way ANOVA (WT: *n* = 4, PBS and LPS: *n* = 6; * *p* < 0.05, *** *p* < 0.001, **** *p* < 0.0001). (**C**) Myd88, IRAK4, and IKKβ mRNA expressions were measured in PBS and LPS groups at 24 h after the treatment. Relative mRNA levels in cauda epididymides were analyzed using quantitative RT-PCR. Data are represented as mean ± SD of three independent experiments and analyzed with independent *t*-test. (PBS and LPS group: *n* = 6; * *p* < 0.05, **** *p* < 0.0001).

In this study, we induced a mouse model of epididymitis via the biliary injection of LPS through the vas deferens close to the cauda epididymis [36]. The same volume of PBS was injected into another group as a control. There were no significant differences of histopathological changes in the LPS and PBS groups at 24 h compared with the WT group (Figure 1A). However, there was considerable inflammation in the cauda epididymis, which was characterized by the infiltration of leukocytes, and interstitial edema after seven days of LPS injection (Figure 1A). After 24 h of the treatment, the expression level of proinflammatory cytokines in the cauda epididymis, such as IL-1α, IL-6, IL-17A, and TNF-α in the LPS group, was significantly increased compared with the WT group and the PBS group (*p* < 0.05, Figure 1B). The other inflammatory cytokines in the cauda at 24 h after the treatment are shown in Figure S3. Seven days after the LPS injection, some inflammatory cytokines could only be detected, in which IL-1α was significantly higher in the LPS group (*p* < 0.05, Figure S4). As LPS can activate both the MYD88-dependent and independent signaling pathways, we also examined the relative expressions of classical genes in the MYD88 signaling pathway in the caput/corpus/caudal area. In the cauda, the expression of Myd88, interleukin-1 receptor-associated kinase 4 (IRAK4), and the inhibitor of nuclear factor kappa-B kinase subunit beta (IKKB) in the LPS group, significantly increased compared with the PBS group, according to real-time PCR (*p* < 0.05, Figure 1C). The relative mRNA expressions of TIR-domain-containing adapter-inducing interferon-β (TRIF) and interferon regulatory factor 3 (IRF3) were also significantly increased in the cauda (*p* < 0.05, Figure S5A). In the corpus, only Myd88 and TRIF were significantly increased (*p* < 0.05, Figure S5B). In the caput, the relative mRNA expressions of Myd88, IRAK4, IKKβ, TRIF, and IRF were significantly increased in the LPS group compared with the PBS group (*p* < 0.05, Figure S5C).

### 2.2. The Inflammatory Infiltrate Was Characterized by the Recruitment of Mo-DCs in LPS-Induced-Epididymitis

**Figure 2:**
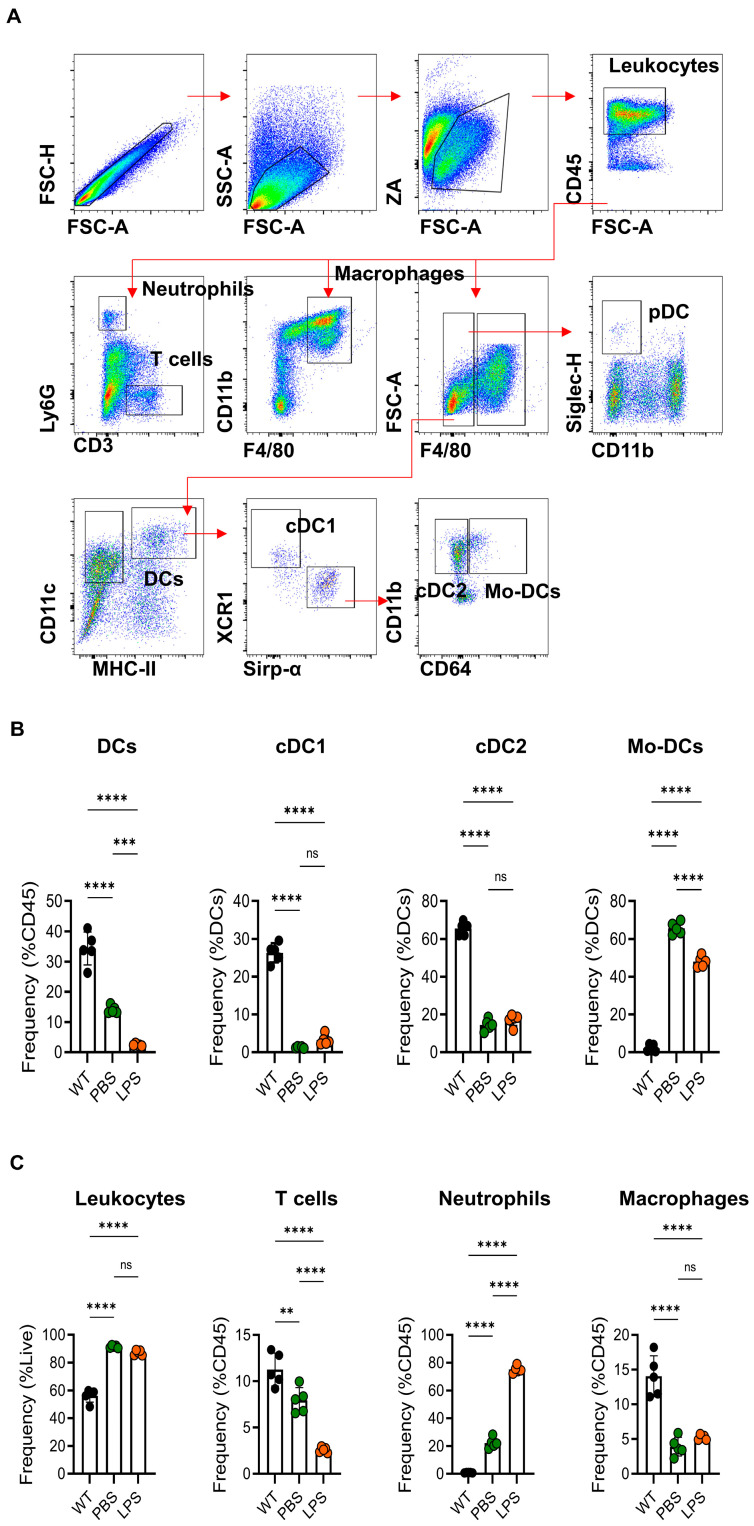
Immune phenotypes of immune cells in LPS-induced mouse epididymitis. (**A**) The gating strategy of epididymal immune cells in flow cytometry. (**B**) Comparison of the frequency of dendritic cell subsets, cDC1, cDC2, and Mo-DCs among three groups. (**C**) The frequencies of CD45^+^ leukocytes, CD3^+^ T cells, Ly6G^+^CD3^−^ neutrophils, and CD11b^+^F4/80^+^ macrophages were analyzed in WT, sham-treated controls, and LPS-induced mice 24 h after treatment via flow cytometry. Data are expressed as mean ± SD and analyzed by one-way ANOVA (*n* = 5), ** *p* < 0.01, *** *p* < 0.001, **** *p* < 0.0001.

The gating strategy of epididymal immune cells in flow cytometry is shown in Figure 2A. After 24 h, the frequencies of Mo-DCs significantly increased in the LPS group compared with the WT group, and similar results were found in the PBS group. However, there was no significant difference in cDC1 and cDC2 (Figure 2B). Furthermore, we also investigated the changes in total leukocytes and subsets, including T cells, neutrophils, and macrophages, 24 h after the treatment. The frequency of leukocytes and neutrophils in the sham and LPS groups increased compared to that in the WT group (Figure 2C). There was no difference in the frequency of leukocytes and macrophages in the LPS and PBS groups. The frequency of neutrophils in the LPS group increased by more than 50 percent over the sham group. In addition, there was a greater reduction in T cells in the LPS group than in the sham group (Figure 2C). Moreover, we also analyzed the variations of these immune cells at day 7 and day 28 after the LPS injection (Figure S6). The total number of leukocytes decreased from day 1 to day 28. The percentage of total DCs, cDC1, cDC2, and T cells, increased gradually from day 1 to day 28, and macrophages increased from day 1 to day 7 but decreased from day 7 to day 28. The percentage of neutrophils and Mo-DCs decreased gradually from day 1 to day 28 (Figure S6).

### 2.3. LPS-Induced Secretion of Proinflammatory Cytokines and Apoptosis in Mouse Bone-Marrow-Derived Dendritic Cells (BMDCs)

**Figure 3:**
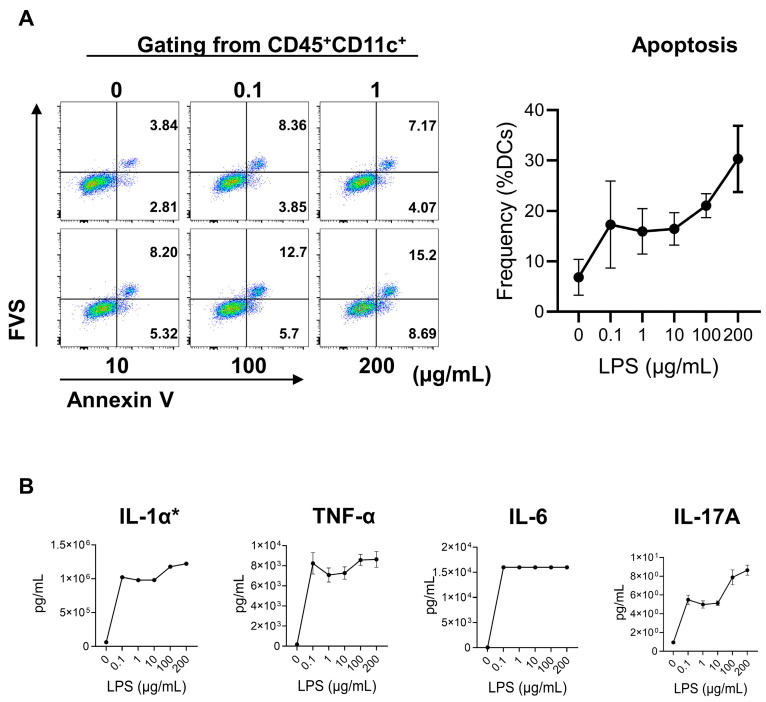
LPS-induced secretion of proinflammatory cytokines and apoptosis of mouse bone-marrow-derived dendritic cells (BMDCs). (**A**) Representative flow cytometry plots and statistics of early and late apoptotic cells (Annexin V^+^) of BMDCs gating from CD45^+^CD11c^+^ under LPS stimulation (0, 0.1, 1, 10, 100, 200 µg/mL; *n* = 3). (**B**) The concentrations of IL-1α, IL-6, IL-17A, and TNF-α in the supernatants at 24 h with 0, 0.1, 1, 10, 100, and 200 µg/mL LPS stimulation (*n* = 3). Results of IL-1α, IL-17A, and TNF-α are expressed as mean ± SD. The lowest concentration of IL-6 after LPS stimulation was over the upper limit of the kit (15,000 pg/mL). * The error bar of IL-1α is too small to plot.

To explore the effect of LPS on the secretion of inflammatory cytokines of DCs in this study, mouse BMDCs were stimulated with different concentrations of LPS for 24 h. The cells were collected for apoptosis analysis via flow cytometry, and supernatants were collected for inflammatory cytokine examination with ELISA. LPS enhanced the apoptosis rate of BMDCs in a dose-dependent manner (Figure 3A). With increasing LPS concentration, the apoptosis frequency of BMDCs increased as well. The apoptosis frequencies were similar at 0.1, 1, and 10 µg/mL (Figure 3A). More than 25% of BMDCs underwent apoptosis under the stimulation of 200 µg/mL LPS. Despite the LPS-induced apoptosis of BMDCs, the concentrations of inflammatory cytokines, including IL-1α, IL-6, IL-17A, and TNF-α significantly increased after LPS stimulation; the concentration of IL-6 was over the highest limit, even under the stimulation of 0.1 µg/mL LPS (Figure 3B). Other inflammatory cytokines that could be detected (IL-12p70 and MCP-1) also increased after LPS stimulation (Figure S7A).

### 2.4. CRISPR-Cas9-Mediated Myd88 Knockout in BMDCs Alleviated the Production of Inflammatory Cytokines

**Figure 4:**
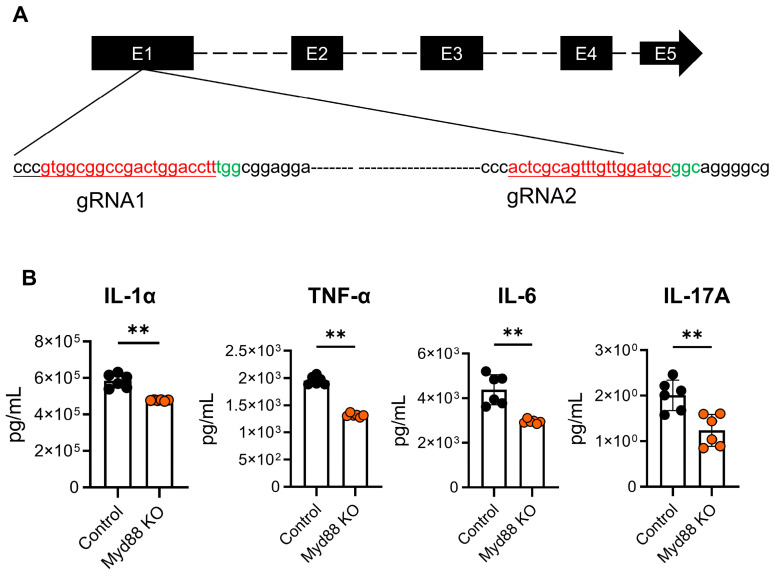
CRISPR-Cas9-mediated Myd88 knockout in BMDCs alleviated the production of proinflammatory cytokines. (**A**) Target sequence of Myd88 gene using Cas9/gRNA system. gRNA-targeting sequences are highlighted in red, and the protospacer adjacent motif (PAM) sequence is highlighted in green. (**B**) The concentrations of IL-1α, IL-6, IL-17A, and TNF-α were detected in Myd88-KO BMDCs and in the control after 24 h with 0.1 µg/mL LPS treatment. Data are expressed as mean ± SD, and statistics were analyzed by independent *t*-test (*n* = 6), ** *p* < 0.01.

To investigate the effect of Myd88 on the secretion of BMDC cytokines, CRISPR-Cas9 was performed to knockout Myd88 in BMDCs. The mice Myd88 gene contains five exons on chromosome 9. The guide RNA1 (gRNA1) and guide RNA2 (gRNA2) were designed downstream of the start codon of Myd88 in exon 1 separately (Figure 4A). After 24 h of stimulation with LPS (1 µg/mL), the supernatant was collected to detect the concentration of inflammatory cytokines by flow cytometry. The concentrations of IL-1α, IL-6, IL-17A, and TNF-α in the Myd88 KO group were significantly suppressed compared with those of the control group (*p* < 0.05, Figure 4B). The expressions of IL-21p70 and MCP-1 were also significantly decreased in the Myd88 KO group (*p* < 0.05, Figure S7B).

### 2.5. CRISPR-Cas9 Knockout Myd88 in DC2s Reduced the Production of Inflammatory Cytokines

**Figure 5:**
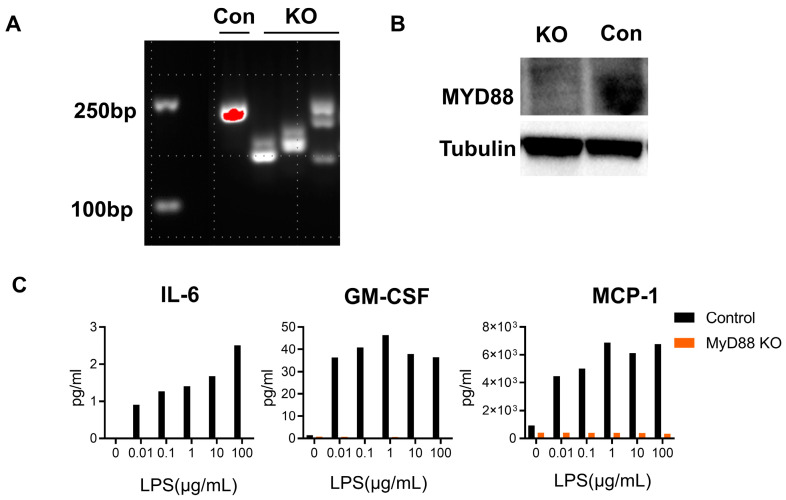
CRISPR-Cas9-mediated Myd88 knockout in mouse distal caput epididymal epithelial cell line (DC2) reduced the production of inflammatory cytokines. DC2s were treated with 0.1 µg/mL LPS for 24 h before DNA/protein sample collection (KO: knockout, Con: control). (**A**) PCR test to confirm the Myd88 expression in Myd88-KO DC2s and control. (**B**) Western blot of MYD88 in DC2s. (**C**): Comparison of the concentrations of IL-6, GM-CSF, and MCP-1 secreted by Myd88-KO DC2s and control with different concentrations of LPS stimulation (0–100 µg/mL) for 24 h. Data are expressed as mean ±SD, and statistics were analyzed by independent *t*-test (*n* = 6).

As mentioned before, the epididymal epithelial cells play an important role in male fertility by supporting sperm motility, maturation, and viability, and also exert an essential role in inflammation induced by LPS 36,37. Therefore, the effect of MYD88 on cytokines secreted by epididymal epithelial cells was investigated, and Myd88 gene knockout in mouse epididymal DC2s was performed in this study. After 48 h of transfection, single cells with GFP gene expression were sorted into a V bottom 96-well plate by flow cytometry. Western blots and PCR were performed to confirm the knockout of the Myd88 gene on the monoclonal strain. The expression of Myd88 decreased according to the PCR results (Figure 5A). Meanwhile, the results of Western blots also confirmed the knockout efficiency (Figure 5B). After 24 h of LPS stimulation, the secretions of IL-6, GM-CSF, and MCP-1 were substantially lower in the KO clone compared with those in the control. (*p* < 0.05, Figure 5C).

## 3. Discussion

Immunological factors in male infertility induced by epididymitis have been found to be an essential factor that cannot be ignored [37,38]. In this study, we used a mouse epididymitis model through the injection of LPS through the vas deferens close to the cauda epididymis. We demonstrated for the first time that (i) LPS induced the recruitment of Mo-DCs and secretion of inflammatory cytokines; (ii) the percentages of resident DCs, macrophages, and T cells significantly decreased in this mouse epididymitis model at 24 h; and (iii) knockout *Myd88* on DCs prevented the release of inflammatory cytokines, which may be helpful for the treatment of epididymitis in the future.

Although there were no significant histopathological changes, the inflammatory cytokines IL-1α, IL-6, IL-17A, and TNF-α and the MYD88 signaling pathway significantly increased compared with the PBS group at 24 h after injection (Figure 1). In addition, the higher levels of the inflammatory cytokines in the LPS group confirmed the successful establishment of the epididymitis model. LPS has been proven to activate the TLR4-MYD88 dependent/independent signaling pathways [39]. We examined the relative mRNA expression of genes involved in the MYD88 signaling pathway and TRIF signaling pathway in the cauda/corpus/caput region of the epididymis. The expression levels of MYD88-related genes increased not only in the cauda region but also in the corpus and caput epididymis, which revealed that the LPS dose used was sufficient to induce inflammation of the whole epididymal segment through MYD88. In our earlier study, we used UPEC to construct the mice model of epididymitis and detected the expression of *Myd88*. There was no significant increase in *Myd88* expression in UPEC-infected epididymitis. We found milder tissue damage, much less fibrosis, and inflammatory response in *Myd88*^−^/^−^ UPEC epididymitis [28]. On the other hand, LPS could be applied to different epididymal regions to study the region-specific inflammatory response [40]. These may explain the different results of the two epididymitis models. It may also relate to the times we detected the expressions of genes, as we detected them at different time points.

Resident DCs play the role of guarders when epididymis is encountered with LPS. Most resident DCs stay immature before they face the pathogens. External antigens rapidly stimulate the maturation of DCs, and then the cytokines (IL-1, IL-6, IL-23, etc.) are released [41,42,43]. We found the frequency of total epididymal DCs sharply decreased at 24 h after LPS injection and recovered gradually over time, but it remained lower until 28 days later. Interestingly, the proportion of cDC1 and cDC2 showed no significant difference between the LPS group and the PBS group. Meanwhile, the frequency of Mo-DCs increased sharply at 24 h post-infection, and gradually decreased from day 1 to day 28 (Figure S6). These results implied that DCs including resident DCs and Mo-DCs had been suffering from exhaustion, especially Mo-DC, derived from blood monocytes. The changes in resident DCs and Mo-DCs suggest that the Mo-DCs may play a more critical role in the response to stimulation with LPS and induce a subsequent immune response.

On the contrary, the frequency of CD3^+^ T cells kept increasing from day 1 to day 28 (Figure S6). The frequency of CD3^+^ T cells was lower in the LPS group at 24 h post-infection, but higher at 28 days post-infection. These results revealed that the T cells received the antigenic signals presented by DCs and were set to work, which has been widely proven [44]. Since the frequency of DCs decreased gradually in the course, the functions of DCs have been investigated, as APCs secreted cytokines to stimulate the differentiation of helper T cells. Sestina Falcone et al. found that LPS at a high concentration can induce the apoptosis of human immature DCs; they performed the test with 0–1000 µg/mL LPS in vitro, and the frequency of apoptotic cells increased sharply at a concentration of 10 µg/mL LPS [45]. Another study also showed a similar result in DC2.4 cells in vitro [46]. Then, we used different concentrations of LPS (0.1 µg/mL to 200 µg/mL) to stimulate the BMDCs in vitro. Our results showed that the apoptosis of BMDCs could be found even at low concentrations of LPS (0.1 µg/mL), and the proportion of apoptotic BMDCs increased with enhanced LPS concentration. Interestingly, the concentration of inflammatory cytokines in supernatants increased under stimulation with LPS (Figure 3). The results of in vitro experiments likely explained the phenomenon in vivo of mouse epididymitis; that is, LPS-induced DCs secrete increased levels of proinflammatory cytokines and prompt them to undergo apoptosis.

As mentioned before, our previous study showed reduced inflammation in *Myd88*^−^/^−^ UPEC epididymitis [28]. However, systemic *Myd88* knockout cannot explain the regional response to infection and molecular mechanisms in the pathogenesis of epididymitis. Previous studies have suggested that *Myd88* played a significant role in the maturation of DCs, although it was not essential for functional DCs maturation [47,48]. Additionally, MYD88 signaling was also significant for DCs to produce proinflammatory cytokines, mediate neutrophil infiltration, and protect against infection [49,50]. On the other hand, MYD88 could regulate the antigen presentation of DCs, and then influence the activation of T cells and B cells [51]. Innate immune activation and T cell polarization required pathogen identification in DCs by MYD88 signaling [22]. Therefore, *Myd88* knockout in DCs may attenuate the function of inducing T cell differentiation, and it may influence the inflammatory status. To explore whether the knockout of *Myd88* on DCs could relieve the inflammation, we designed gRNAs that targeted exon 1 of *Myd88*. As the number of DCs sorted from the epididymis of mice model were too small to perform the experiment, BMDCs were selected for the in vitro experiments. Our results demonstrated the reduced secretion of inflammatory cytokines in BMDCs after the knockout of *Myd88*, which is consistent with findings of previous research [52,53]. The next step was to verify this effect through a specific knockout of *Myd88* on DCs or monocytes in vivo. Moreover, we conducted the knockout of *Myd88* on DC2s to explore the role of epithelial cells in inflammation. Only three kinds of inflammatory cytokines (IL-6, GM-CSF, MCP-1) could be detected in the supernatants after stimulation with LPS on DC2s. All of these cytokines were reduced after the knockout *Myd88* on DC2s. The results showed that the epithelial cells are also partly involved in the inflammatory response induced by LPS in the epididymis of mice.

There are some limitations in this study. In vivo, we did not show the expression of *Myd88* in epididymal DCs because of some technical problems. The number of epididymal DCs per mouse in the LPS group was too low to conduct RNA isolation. Even if we had pooled six mice together to sort epididymal DCs and then used the RNAprep Pure Micro Kit (TIANGEN, Beijing, China, Cat. No. 4992859) to extract total RNA, we still could not obtain enough RNA for qPCR experiments. Furthermore, we did not perform the effect of *Myd88*-conditional KO in DCs in vivo. Although our previous study has demonstrated the reduction of inflammation in *Myd88^−^/^−^* mice, the effect of gene-editing targets on epididymal DCs is still unclear. Further studies would be needed for verification.

In summary, we found that the MYD88 signaling pathway could modulate the secretion of inflammatory cytokines in DCs, which has an essential role in the pathogenesis of epididymitis. Applying the CRISPR/Cas9-mediated *Myd88* knockout on BMDCs can decrease the concentration of inflammatory cytokines. These findings may help to provide new insights into the underlying mechanism and treatment of epididymitis and male infertility.

## 4. Materials and Methods

### 4.1. Animals

All animal experiments were approved by the Institutional Review Board (IRB) of the University of Hong Kong-Shenzhen Hospital (HKUSZH2020014). C57BL/6J mice (10–12 weeks old) were purchased from Guangdong Vital River Laboratory Animal Technology Co., Ltd. The mice were bred under a controlled 12/12 h light/dark cycle and temperature (22–24 °C), with food and water at liberty.

### 4.2. Experimental Design

The C57 mice were anesthetized by the inhalation of isoflurane, and then processed by retrograde injection of 5 μL of sterile solution containing 25 μg of LPS or phosphate-buffered saline (PBS), via the vas deferens, into the epididymis [33]. The LPS dose was equivalent to 25,000 (25 μg) endotoxin units (EU), according to the manufacturer’s manual (*E. coli* O111: B4, Sigma, Fukushima, Japan). The mice were euthanized at 1 day and 7 days after LPS injection. The mice were divided into three groups: the LPS group, the PBS group, and the wild-type group (WT, untreated control).

### 4.3. Histopathological Evaluation

Mice epididymal tissues were fixed in Bouin’s solution for 24 h. Sections were stained with hematoxylin/eosin, and then analyzed for histopathological changes using an Olympus Slideview VS2000 microscope (Olympus Corporation, Tokyo, Japan).

### 4.4. Quantitative Real-Time RT–PCR

Total RNA from mice caput/cauda/corpus epididymis were extracted using the total RNA solation kit (Vazyme, China, Cat. No. RC112-01). cDNA was synthesized from total RNA using the all-in-one RT SuperMix kit (Vazyme, Nanjing, China, Cat. No. R333-01). cDNA was amplified using the Taq Pro Universal SYBR qPCR Master Mix kit (Vazyme, Nanjing, China, Cat. No. Q712-02), with the primers shown in Supplementary Materials Table S1. The amplification was performed in an ABI PRISM 7500 Real-Time PCR system (Applied Biosystems, Merritt, Norwalk, CT, USA). The relative fold change of all target genes was normalized to GAPDH by calculating 2^-ΔCt^.

### 4.5. Epididymal Immune Cell Suspension Isolation

After sacrificing the mice, all of the epididymal tissues were separated, and the adipose tissue was carefully removed. The bilateral epididymides of each mouse were placed into a 1.5 mL conical tube containing 1 mL of RPMI 1640 medium with 5% fetal bovine serum (FBS, ThermoFisher, Waltham, MA, USA, Cat. No. 10091148), and then mechanically dissociated by cutting with microscopic scissors. The epididymal tissue was washed twice with cold PBS to remove as much sperm as possible, and then placed in a 15 mL conical tube with 4 mL of RPMI 1640 medium containing collagenase IV (1 mg/mL, ThermoFisher, Waltham, MA, USA, Cat. No. 17104019), DNase I (0.1 mg/mL, Merck, Lebanon, NJ, USA, Cat. No. 11284932001), and 20% FBS. The tube was shaken in a 45-degree position at 220 rpm and 37 °C for 40 min. At the end of incubation, 8 mL of RPMI 1640 medium containing 10% FBS was added to stop the digestion. The digested suspensions were filtered through a 40 µm cell strainer before centrifugation at 400× *g* for 6 min at 4 °C. The cells were resuspended in 5 mL of 40% percoll (Merck, Lebanon, NJ, USA, Cat. No. GE17-0891-01), followed by density gradient centrifugation (500× *g* for 25 min at 20 °C, acc/dec: 3/2). The mononuclear cell layer was isolated, collected in a new tube, and rinsed with 0.9% NaCl. The cells were prepared for flow cytometer staining.

### 4.6. Flow Cytometry Analysis of Epididymal Immune Cells

For the epididymal immune cells, the cell suspension was incubated with CD16/32 (Biolegend, San Diego, CA, USA, Cat. No. 101302) to block Fc receptors, and initially stained for viability using Zombie Aqua (Biolegend, San Diego, CA, USA, Cat. No. 423101) at 4 °C for 15 min. After washing once with PBS, the cells were then stained using fluorochrome-conjugated monoclonal antibodies, as supplied in Table S2. The cells were washed with PBS again, and then resuspended in FACS buffer and examined with a FACS analyzer (Beckman Coulter CytoFLEX™, Brea, CA, USA).

### 4.7. Cell Culture

As previously described, bone marrow cells were separated from the femur and tibia of 10-week-old male C57BL/6J mice [34,35]. Briefly, the femurs and tibias were flushed with a 1 mL sterile syringe containing 1 mL of RPMI 1640 medium (5% FBS). The cell suspension was filtered with a 70 μm filter and centrifuged (500× *g* for 5 min). The cells were then lysed with 1 mL (per mouse) of red blood cell lysate (Solarbio, Beijing, China, Cat. No. R1010) on ice for 3 min, and this was stopped by adding 10 mL of PBS. The cells were washed once with PBS and resuspended with complete medium after centrifugation (500× *g* for 5 min). The cells were re-suspended at 1 × 10^6^ per mL of complete medium (10% FBS, 100 U/mL penicillin/streptomycin and 1 mM sodium pyruvate in RPMI 1640 medium) containing 20 ng/mLGM-CSF and 15 ng/mL IL-4 after being washed once with PBS. On day 2 and day 4, 3/4 of the medium was removed and supplied with an equal volume of fresh complete medium. On day 6, suspension cells were collected and adherent cells were loosened for an additional two days of culture. On day 8, LPS was added to stimulate immature BMDCs. On day 9, the cells or supernatant were collected for detection. The generation of BMDCs was confirmed via morphology and flow cytometry analysis (Supplementary Materials Figure S1).

As the epididymal epithelium plays an important role in male fertility by supporting sperm motility, maturation, and viability, the immortalized mouse epididymal (DC2) cell line was performed as the control. The DC2s were kindly provided by Dr. Winnie Shum from ShanghaiTech University, China. The cells were cultured in IMDM (ThermoFisher, Waltham, MA, USA, Cat. No. 12440053) containing 10% FBS, 100 U/mL penicillin/streptomycin, 1 mM sodium pyruvate, and 1 nM dihydrotestosterone (Selleck, Houston, TX, USA, Cat. No. S4757). LPS was added to stimulate DC2 for 24 h, and the cells or supernatant were collected for detection.

### 4.8. Detection of BMDCs Apoptosis

BMDCs were collected after 24 h stimulation with LPS and then stained at 4 °C for 30 min with surface markers and Fixable Viability Stain (BD, Franklin Lakes, NJ, USA, Cat. No. 564406). After being washed once with PBS, the BMDCs were then stained with Annexin V (Biolegend, San Diego, CA, USA, Cat. No. 640912) at room temperature for 15 min and were washed once with Annexin V binding buffer. Finally, the BMDCs were resuspended in 200 μL of FACS buffer and examined with a FACS analyzer (Beckman Coulter CytoFLEX™, Brea, CA, USA).

### 4.9. CRISPR-Cas9 Interference of Myd88

The plasmids containing the single-guide RNA target on Myd88 and EGFP gRNA were designed and synthesized by Syngentech (Beijing, China). The BMDCs (day 5 of culture) and DC2s were transfected with control gRNA (g0) or target gRNA (g1 or g2) using a transfection reagent (Polyplus Transfection, Illkirch, France, Cat. No. 101000046), according to the manufacturer’s instructions. For BMDCs, the cells were cultured and collected as described above. For DC2s, after 24 h of transfection, the medium was removed, and fresh medium was supplied to enhance the frequency of live cells. After 48 h of transfection, the DC2s were collected and stained with Zombie Aqua to define live/dead cells for single-cell sorting. Single cells with Zombie Aqua-negative and GFP-positive were sorted into a 96-well plate, and then cultured for about 15 days. The cells were transferred into a 6-well plate after single-cell clones were observed. The efficiency of the knockout was confirmed via PCR and Western blot. The gating strategy for cell sorting is shown in Supplementary Materials Figure S2A.

### 4.10. Bead-Assisted Multiplex Cytokine Profiling

The total protein isolated from mouse epididymal cauda, supernatant of BMDCs, and DC2s after LPS stimulation for 24 h was prepared for inflammatory cytokine examination. A mouse inflammation panel (13-plex) was performed to detect the concentrations of cytokines using a bead-assisted multiplex cytokine profiling kit (Biolegend, San Diego, CA, USA, Cat. No. 740446). Briefly, tissues or supernatant concentrations of interleukin-1α (IL-1α), interleukin-23 (IL-23), interferon-γ (IFNγ), tumor necrosis factor-α (TNF-α), monocyte chemoattractant protein-1 (MCP-1), interleukin-12p70 (IL-12p70), interleukin-1β (IL-1β), interleukin-10 (IL-10), interleukin-6 (IL-6), interleukin-27 (IL-27), interleukin-17A (IL-17A), interferon-β (IFN-β), and granulocyte-macrophage colony-stimulating factor (GM-CSF) were simultaneously detected following the manufacturer’s instructions. The samples were read on a flow cytometer (Beckman Coulter CytoFLEX™, Brea, CA, USA) and the data were analyzed through an online tool supplied by Biolegend (legendplex.qognit.com (accessed on 20 December 2021)).

### 4.11. Western Blot

The DC2s were collected and lysed using lysis buffer (Beyotime Biotechnology company, Shanghai, China, Cat. No. P0013) with a protease inhibitor cocktail (Beyotime Biotechnology company, Shanghai, China, Cat. No. P1006) at 4 °C for 10 min. Equal amounts of protein (20 µg) were loaded onto 10% precast gels (GenScript, Nanjing, China, Cat. No. M00666), and then transferred onto nitrocellulose membranes. The membranes were blocked with block buffer (Beyotime, Shanghai, China, Cat. No. P0239) for 30 min, and then incubated overnight at 4 °C with primary antibodies anti-Myd88 (1:500, Arigo, Taiwan, China, Cat. No. ARG41275) and anti-beta tubulin antibody (1:1000, Abcam, Cambridge, UK, Cat. No. ab6046). Relevant horseradish peroxidase-conjugated secondary antibodies were applied for 1 h after washing three times in PBST. The expressions of proteins were observed using the Molecular Imager ChemiDoc XRS+ system (Bio-Rad, Hercules, CA, USA). The image was cropped for presentation, and the full-length image is supplied in Figure S2B.

### 4.12. PCR

The total DNA of DC2s was extracted using a genomic DNA extraction kit (Accurate biotechnology, Danyang, China, Cat. No. AG21009) according to the manufacturer’s instructions. A total of 400 ng of DNA were amplified using the 2X Accurate Taq Master Mix kit (Accurate biotechnology, Danyang, China, Cat. No. AG11009) with the following PCR conditions: 94 °C for 30 s followed by 40 cycles (98 °C for 10 s, 55 °C for 30 s, and 72 °C for 1 min), and then 72 °C for 2 min. The products were subjected to gel electrophoresis, and then the bands were photographed under the Molecular Imager ChemiDoc XRS+ system (Bio-Rad, Hercules, CA, USA).

## Data Availability

The data presented in this study are available on request from the corresponding author.

## Supplementary Materials

The following supporting information can be downloaded at: https://www.mdpi.com/article/10.3390/ijms24097838/s1.
