# Supplementary material for: Myd88 Signaling Is Involved in the Inflammatory Response in LPS-Induced Mouse Epididymitis and Bone-Marrow-Derived Dendritic Cells
Source: Int J Mol Sci. 2023 Apr 25;24(9):7838. doi: 10.3390/ijms24097838 (PMC10178089; doi:10.3390/ijms24097838)
Supplement: Supplementary file 1 [file ijms-24-07838-s001.zip › ijms-2294968 Supplemental figures.pdf]

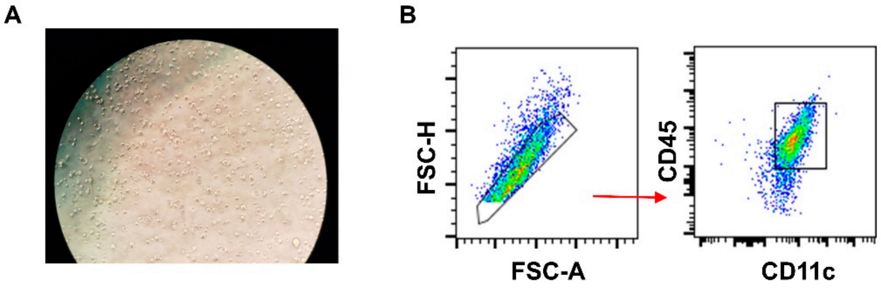

Figure S1. Identification of BMDCs. **A.** Morphology of BMDCs on day 8. **B.** Flow cytometry analysis of phenotype of BMDCs (CD45$^+$CD11C$^+$, gating from live cells).

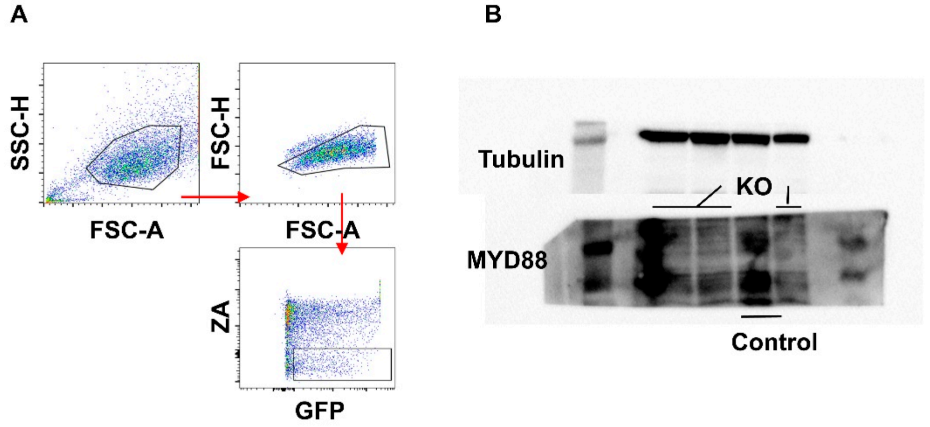

Figure S2. Representative flow cytometry plots of sorting DC2s and original image of western blot. **A.** Representative flow cytometry plots of gating strategy for sorting DC2s which demonstrated successful transfection with plasmid containing *Myd88* gRNA. **B.** Original image of western blot showed MYD88 expression in *Myd88*-KO DC2s and control.

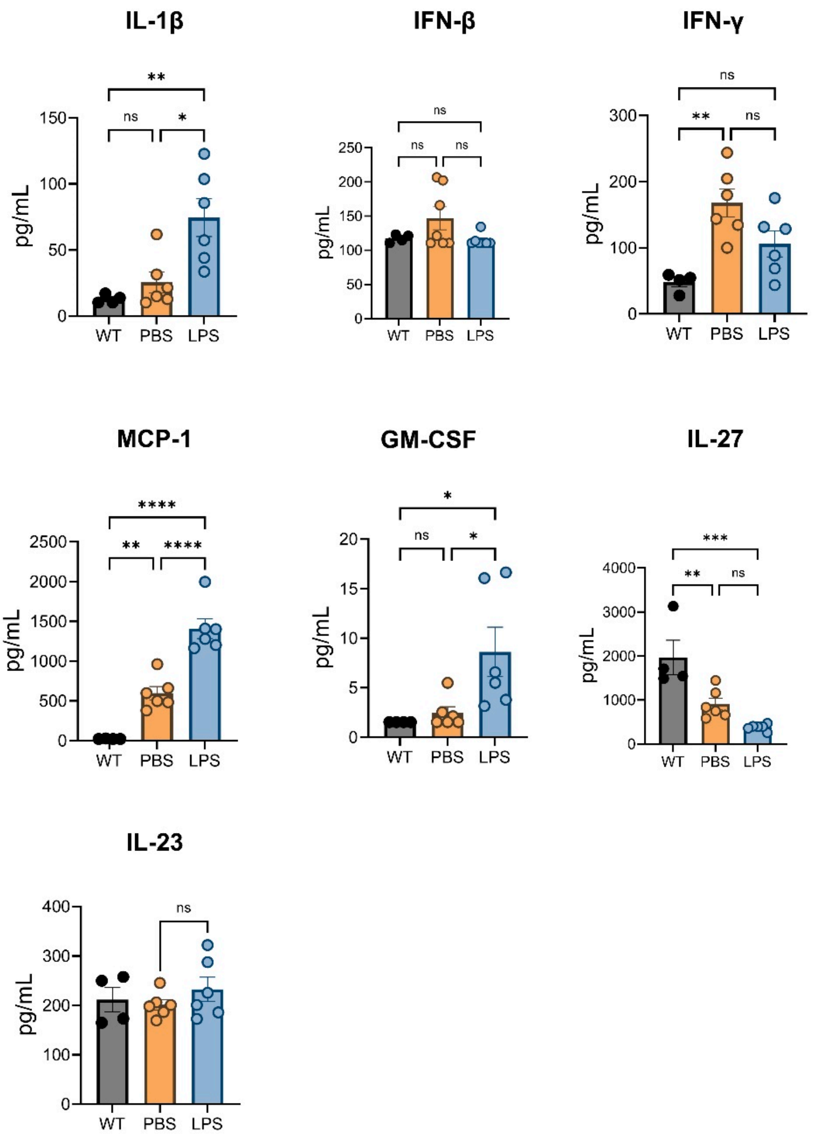

Figure S3. Comparison of other inflammatory cytokines detected in cauda epididymis 24 hours after LPS treatment. The concentration of IL-1β, IFN-β, IFN-γ, MCP-1, GM-CSF, IL-27, and IL-23 was measured in wild type (WT), sham treated controls (PBS), and LPS-induced mice (WT: n=4, PBS and LPS: n=6). Results were expressed as mean ± SD and were analyzed by one-way ANOVA. *$p<0.05$, **$p<0.01$, ***$p<0.001$, ****$p<0.0001$; ns, not significant.

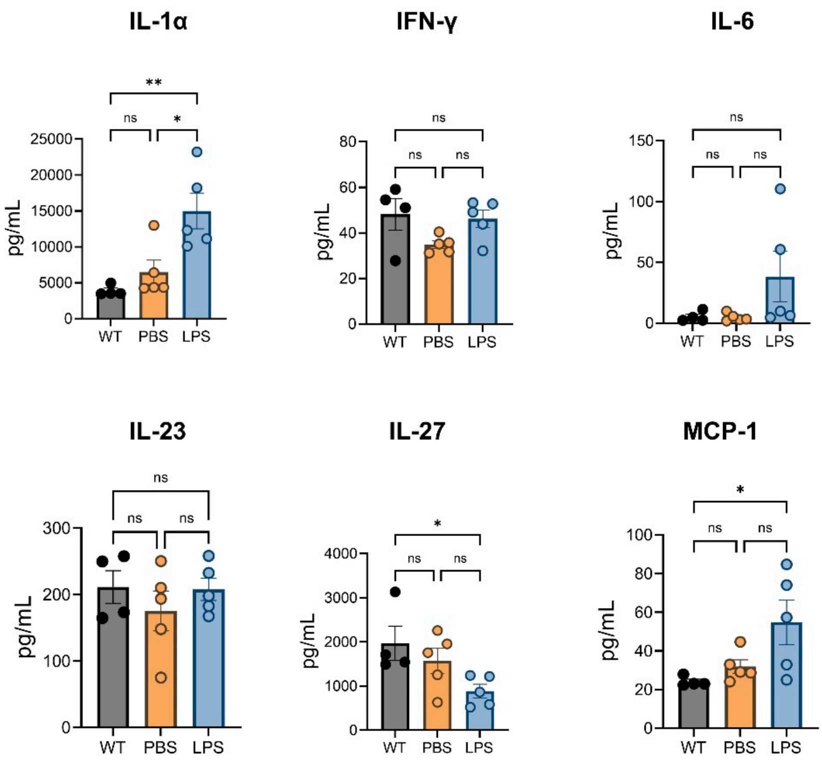

Figure S4. Inflammatory cytokines detected in cauda epididymis at 7 days after LPS treatment. Comparison of IL-α, IFN-γ, IL-6, IL-23, IL-27, and MCP-1 in WT, PBS, and LPS-induced mice (WT: n=4, PBS and LPS: n=5). Results are expressed as mean ± SD and were analyzed by one-way ANOVA. *p<0.05, **p<0.01; ns, not significant.

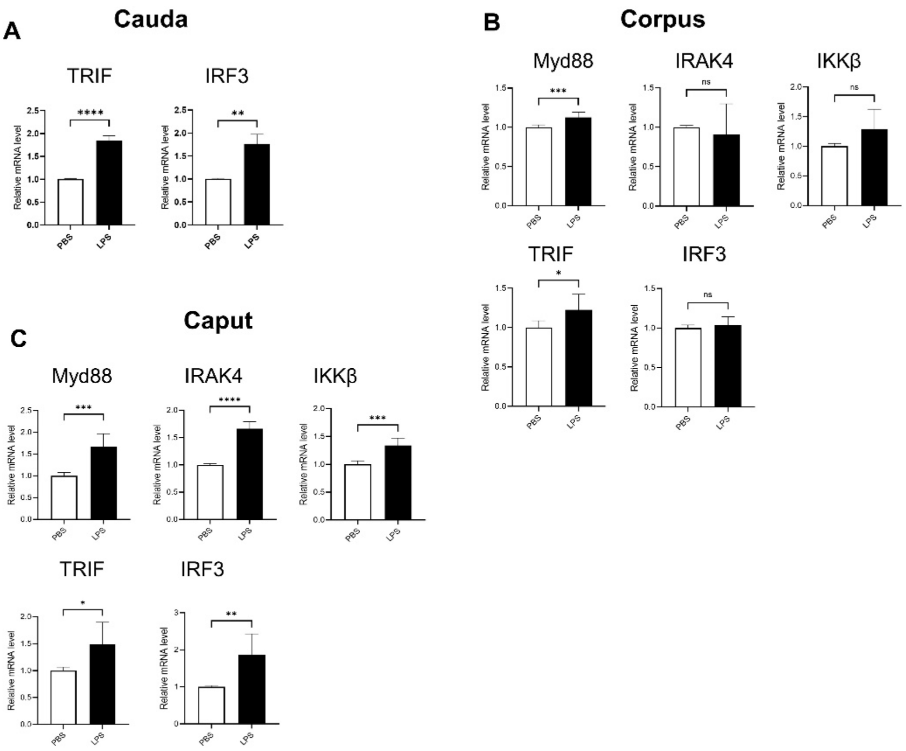

Figure S5. The mRNA of *Myd88*/*TRIF* signaling pathway expressed in caput, corpus, and cauda in LPS-induced mouse epididymitis. **A**. Analysis of relative mRNA expression of *TRIF* and *IRF3* in cauda epididymis between PBS and LPS groups. **B-C**: Comparison of relative mRNA expression of *MyD88, IRAK4, IKKβ, TRIF* and *IRF3* in corpus (**B**) and caput (**C**) and epididymis between PBS and LPS group. Data were represented as mean ± SD of three independent experiments and analyzed by independent t-test. *p<0.05, **p<0.01, ***p<0.001, ****p<0.0001; ns, not significant.

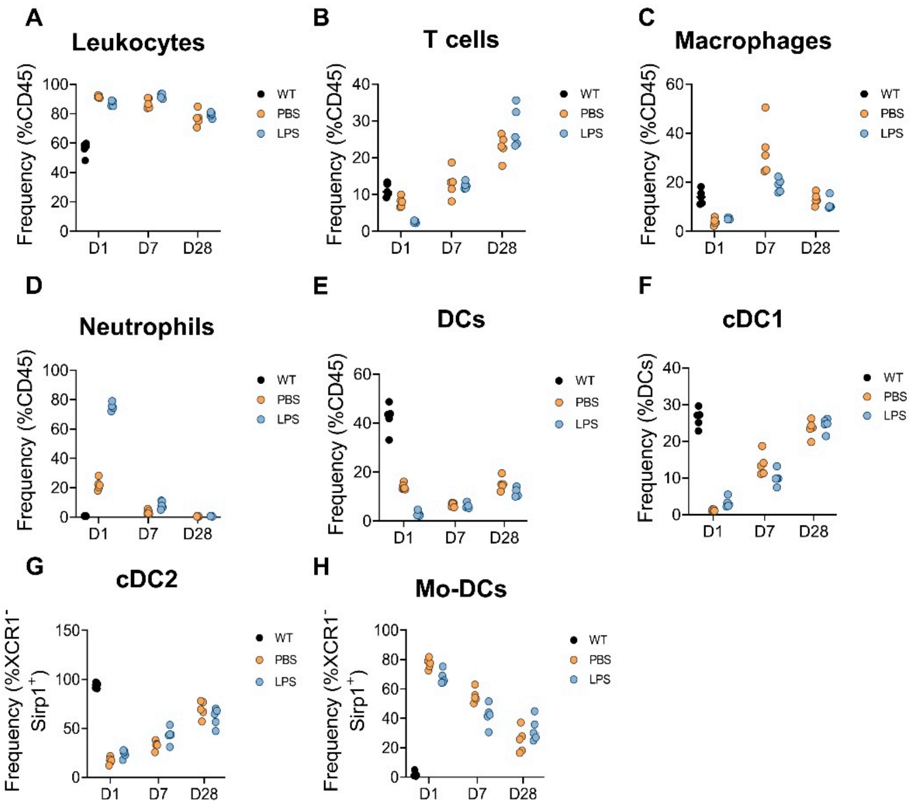

Figure S6. The percentage of immune cells in LPS-induced mouse epididymitis at different time points. **A-D**: The percentage of epididymal leukocytes, T cells, macrophages and neutrophils was detected in WT, PBS, and LPS groups at 1, 7, 28 days after treatment (n=5). **F-H:** The proportion of epididymal DCs and subsets was measured at the indicated time point (n=5).

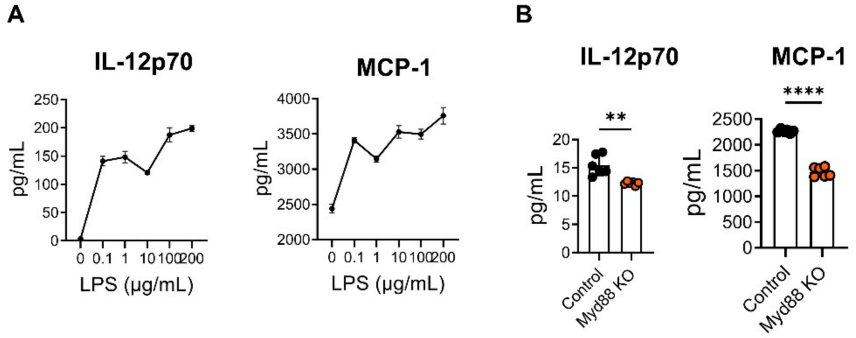

Figure S7. Other Inflammatory cytokines in mouse bone marrow-derived dendritic cells (BMDCs) with CRISPR-Cas9 mediated *Myd88* knockout. **A.** The concentration of IL-12p70 and MCP-1 in the supernatants after 24 hours with 0-0.1-1-10-100-200 μg/ml LPS stimulation (n=3). **B.** Comparison of IL-12p70 and MCP-1 secreted by *Myd88*-KO BMDCs and control were analyzed. Data were expressed as mean ± SD and statistics were analyzed by independent t test (n=6), **p<0.01, ****p<0.0001.
