# Supplementary material for: Myd88 Signaling Is Involved in the Inflammatory Response in LPS-Induced Mouse Epididymitis and Bone-Marrow-Derived Dendritic Cells
Source: Int J Mol Sci. 2023 Apr 25;24(9):7838. doi: 10.3390/ijms24097838 (PMC10178089; doi:10.3390/ijms24097838)
Supplement: Supplementary file 1 [file ijms-24-07838-s001.zip › ijms-2294968 Supplemental tables.pdf]

Table S1. Primers used for PCR and RT-PCR.

Species	Primer name	Forward primer (5′-3′)	Reverse primer (5′-3′)
mouse	*Gapdh*	CTGACTTCAACAGCGACACC	TAGCCAAATTCGTTGTCATACC
	Myd88	ATGGTGGTGGTTGTCTCTGA	TGCTGGGGAACTCTTTCTTC
	Myd88[#]	GCGCTTAACGTGGGAGTG	ATATCCTCACGGTCTAACAAGG
	Irak4	GGTCCAGATTGAGCTGTTTGCC	GTTTGTGCCACTGTTGCCGCTT
	Ikkβ	GCAGACTGACATTGTGGACCTG	ATCTCCTGGCTGTCACCTTCTG
	Trif	ATCCATGCCAGGGCTGATGAAC	CGATGGCATCTTGGAGACAGTG
	Irf3	CGGAAAGAAGTGTTGCGGTTAGC	CAGGCTGCTTTTGCCATTGGTG

[#]Primer used for PCR

Table S2 List of antibodies used for flow cytometry analysis.

Antibodies	Source	Clone	Cat.No
R718 Rat Anti-Mouse CD45	BD	I3/2.3	567464
PE-CF594 Hamster Anti-Mouse CD3e	BD	145-2C11	562286
PE-Cy™7 Rat Anti-Mouse Ly-6G	BD	1A8	560601
BV650 Rat Anti-CD11b	BD	M1/70	563402
Brilliant Violet 605™ anti-mouse F4/80	BioLegend	BM8	123133
SIGLEC H Monoclonal Antibody (eBio440c), PE	eBioscience	eBio440c	12-0333-82
BV421 Armenian Hamster Anti-Mouse CD11c	BD	N418	565452
BB515 Rat Anti-Mouse I-A/I-E	BD	2G9	565254
APC/Cyanine7 anti-mouse/rat XCR1 Antibody	Biolegend	ZET	148224
APC Rat anti-Mouse CD172a	BD	P84	560106
BV786 Mouse Anti-Mouse CD64 a and b Alloantigens	BD	X54-5/7.1	741024
